# Online-Mediated HIV Pre-exposure Prophylaxis Care and Reduced Monitoring Frequency for Men Who Have Sex With Men: Protocol for a Randomized Controlled Noninferiority Trial (EZI-PrEP Study)

**DOI:** 10.2196/51023

**Published:** 2023-11-08

**Authors:** Marije L Groot Bruinderink, Anders Boyd, Liza Coyer, Sophie Boers, Laura Blitz, Jean-Marie Brand, Hannelore M Götz, Martijn Stip, Joey Woudstra, Kenneth Yap, Koenraad Vermey, Amy Matser, Allard R Feddes, Vita W Jongen, Maria Prins, Elske Hoornenborg, Frenk van Harreveld, Maarten F Schim van der Loeff, Udi Davidovich

**Affiliations:** 1 Department of Infectious Diseases Public Health Service of Amsterdam Amsterdam Netherlands; 2 Department of Psychology University of Amsterdam Amsterdam Netherlands; 3 HIV Monitoring Foundation Amsterdan Netherlands; 4 Department of Sexual Health Public Health Service of Gelderland-Zuid Nijmegen Netherlands; 5 Department of Sexual Health Public Health Service of Haaglanden The Hague Netherlands; 6 Department of Infectious Diseases Public Health Service of Rotterdam-Rijnmond Rotterdam Netherlands; 7 Department of Public Health Erasmus MC University Medical Center Rotterdam Netherlands; 8 Aidsfonds – Soa Aids Nederland Amsterdam Netherlands; 9 Amsterdam Institute for Infection & Immunity Department of Infectious Diseases Amsterdam UMC, University of Amsterdam Amsterdam Netherlands; 10 National Institute for Public Health and the Environment Bilthoven Netherlands

**Keywords:** pre-exposure prophylaxis, randomized controlled trial, telemedicine, HIV prevention, MSM, men who have sex with men, Netherlands, adherence, acceptability, usability

## Abstract

**Background:**

Daily and event-driven HIV pre-exposure prophylaxis (PrEP) with oral tenofovir-emtricitabine is highly effective to prevent HIV in men who have sex with men (MSM). PrEP care generally consists of in-clinic monitoring every 3 months that includes PrEP dispensing, counseling, and screening for HIV and sexually transmitted infections (STIs). However, the optimal frequency for monitoring remains undetermined. Attending a clinic every 3 months for monitoring may be a barrier for PrEP. Online-mediated PrEP care and reduced frequency of monitoring may lower this barrier.

**Objective:**

The primary objective of this study is to establish the noninferiority of online PrEP care (vs in-clinic care) and monitoring every 6 months (vs every 3 months). The secondary objectives are to (1) examine differences between PrEP care modalities regarding incidences of STIs, HIV infection, and hepatitis C virus infection; retention in PrEP care; intracellular tenofovir-diphosphate concentration; and satisfaction, usability, and acceptability of PrEP care modalities; and (2) evaluate associations of these study outcomes with sociodemographic, behavioral, and psychological characteristics.

**Methods:**

This study is a 2×2 factorial, 4-arm, open-label, multi-center, randomized, controlled, noninferiority trial. The 4 arms are (1) in-clinic monitoring every 3 months, (2) in-clinic monitoring every 6 months, (3) online monitoring every 3 months, and (4) online monitoring every 6 months. The primary outcome is a condomless anal sex act with a casual partner not covered or insufficiently covered by PrEP (ie, “unprotected act”) as a proxy for HIV infection risk. Eligible individuals are MSM, and transgender and gender diverse people aged ≥18 years who are eligible for PrEP care at 1 of 4 participating sexual health centers in the Netherlands. The required sample size is 442 participants, and the planned observation time is 24 months. All study participants will receive access to a smartphone app, which contains a diary. Participants are requested to complete the diary on a daily basis during the first 18 months of participation. Participants will complete questionnaires at baseline and 6, 12, 18, and 24 months. Dried blood spots will be collected at 6 and 12 months for assessment of intracellular tenofovir-diphosphate concentration. Incidence rates of unprotected acts will be compared between the online and in-clinic arms, and between the 6-month and 3-month arms. Noninferiority will be concluded if the upper limit of the 2-sided 97.5% CI of the incidence rate ratio is <1.8.

**Results:**

The results of the main analysis are expected in 2024.

**Conclusions:**

This trial will demonstrate whether online PrEP care and monitoring every 6 months is noninferior to standard PrEP care in terms of PrEP adherence. If noninferiority is established, these modalities may lower barriers for initiating and continuing PrEP use and potentially reduce the systemic burden for PrEP providers.

**Trial Registration:**

ClinicalTrials.gov NCT05093036; https://tinyurl.com/28b8ndvj

**International Registered Report Identifier (IRRID):**

DERR1-10.2196/51023

## Introduction

Oral tenofovir (TFV) disoproxil combined with emtricitabine (FTC) is highly efficacious as pre-exposure prophylaxis (PrEP) for the prevention of HIV infection [1,2]. PrEP can be taken daily [3-5] or in an event-driven schedule by cisgender men or transgender and gender diverse people (TGDP) assigned male at birth, who are not taking exogenous estradiol-based hormones [6-8]. Globally, the standard of care generally entails in-clinic PrEP initiation and in-clinic monitoring visits every 3 months, including PrEP prescription and dispensing, counseling on PrEP adherence and other HIV prevention strategies, and HIV testing, and may include kidney function testing [8,9]. PrEP programs are recommended to integrate PrEP monitoring visits with testing for bacterial sexually transmitted infections (STIs) and for hepatitis B virus and hepatitis C virus (HCV) infections [8-12]. If combined with the recommended HIV testing frequency, such testing is commonly performed every 3 months, although in practice, the frequency of testing could depend on sexual behavior [8-12].

In-clinic PrEP care faces several challenges that stem from the recommended frequency of monitoring visits (every 3 months). Clients might have to take time off from work, school, or family obligations, and may incur travel costs for attending in-clinic visits [13-17]. Persons interested in PrEP may also feel burdened with perceived stigma [18,19], cultural or social insensitivity [16,20,21], and lack of privacy [14,15] at the clinic. Providers might have capacity problems at facilities owing to shortages of health care staff or budget constraints, and might be forced to limit the number of clients or prioritize certain clients [22-24]. These client and provider barriers may contribute to individuals not initiating PrEP or discontinuing PrEP, which in turn increases the risk of avoidable HIV infections [23,25].

Limiting the number of in-clinic visits by offering online-mediated PrEP care may address some of these barriers [8,26,27]. Different models of digital PrEP services had been implemented across income settings prior to the COVID-19 pandemic, and implementation accelerated since the start of the pandemic [8,26-29]. Online PrEP care generally includes a combination of the following components: telephone or video consultation to collect data regarding medical history, sexual behavior, and PrEP intake, which are needed for PrEP prescription and counseling [8,26-29]; specimen collection for HIV and STI testing at a local laboratory [30], at home using a self-sampling kit [31,32], or a combination of both [30,33]; and delivery of PrEP tablets at locations preferred by users (eg, at home, other addresses, or health facilities) [30,33]. Since online PrEP care involves less intensive contact between PrEP users and health care providers and thus less guidance on PrEP use, it could theoretically have an adverse effect on PrEP adherence. Studies so far have shown high acceptability of online PrEP services among PrEP users. However, these studies were small in scale and were predominantly descriptive in nature and thus did not provide rigorous evidence on PrEP adherence in comparison to the standard of care [29,34], with the exception of an ongoing randomized controlled trial in the United States [31]. In their study, the authors aimed to compare online PrEP care with the standard of care with respect to protective levels of tenofovir diphosphate (TFV-DP) in dried blood spots (DBSs), and the results of this study are still pending [31]. Other trials related to online PrEP care in the European context do not yet exist.

Limiting the number of in-clinic visits through a reduction in the frequency of PrEP monitoring may pose challenges for the timely screening of HIV and other STIs. Regular HIV testing during PrEP use is essential to identify breakthrough HIV infections in suboptimally adherent PrEP users and, rarely, in highly adherent PrEP users [35,36]. It allows for commencing HIV treatment as soon as possible and avoiding onward transmission and the development or transmission of drug-resistant strains. While the current guidelines recommend HIV testing every 3 to 6 months [8-12], few studies have formally investigated the impact of HIV screening frequency on PrEP adherence [8]. A lower frequency of contact moments and HIV testing may negatively affect PrEP intake. One randomized noninferiority trial in Kenya found similar PrEP adherence in adults with dispensing of PrEP every 6 months at the clinic combined with home-sampled HIV testing at 3 months after dispensing and in adults with dispensing of PrEP and HIV testing every 3 months at the clinic as the standard of care [37]. More evidence from other regions and from different groups of PrEP users is needed to further evaluate the implications of reduced frequency of monitoring. Particularly in high income settings with low HIV prevalence and high community-level PrEP uptake and PrEP adherence, HIV testing every 6 months might be sufficient. PrEP monitoring visits for men who have sex with men (MSM) in these settings are commonly combined with STI testing, but the optimal frequency of STI screening among MSM who use PrEP is unknown [2,22,38]. On the one hand, reducing the frequency of PrEP monitoring might lead to delayed diagnosis and treatment of asymptomatic STIs and onward transmission. On the other hand, it might avoid antibiotic use related to asymptomatic chlamydia and gonorrhea [39-41], and a lower STI screening frequency might be sufficient for MSM with lower levels of sexual activity.

The *E-Health for Zero Infections - facilitating access to and use of Pre-Exposure Prophylaxis in the Netherlands* (EZI-PrEP) study has been designed to assess whether online-mediated PrEP care and a reduced frequency of monitoring are noninferior compared to standard practice. The secondary research aims include assessing the effect of reduced monitoring frequency on HIV and STI testing behaviors and assessing whether the frequency of testing matches the level of behaviors associated with acquiring these infections. In the EZI-PrEP study, we will develop, implement, and evaluate an online-mediated PrEP care service and a less frequent monitoring schedule (every 6 months) among MSM and TGDP in 4 regions in the Netherlands. This paper describes the design and methods of the EZI-PrEP study.

## Methods

### Study Objectives

The primary objective of the EZI-PrEP study is to assess whether online-mediated PrEP care is noninferior to standard in-clinic care and whether monitoring every 6 months is noninferior to standard monitoring every 3 months. We defined the primary outcome measure as a condomless anal sex act with a casual partner during which PrEP was not used or insufficiently used (herein referred to as an “unprotected act”). Ideally, incident HIV infection would be the primary outcome, but this outcome is expected to be exceedingly rare for use in a clinical trial [42]. The outcome “unprotected act” can be regarded as a behavior that carries the most risk for HIV acquisition and is expected to occur frequently enough to serve as the primary outcome.

The secondary objective is to compare differences between PrEP care modalities in the incidences of bacterial STIs (ie, chlamydia, gonorrhea, and syphilis), HIV infection, and HCV infection; intracellular concentrations of TFV-DP; retention in PrEP care; total number of STI consultations (in addition to PrEP monitoring visits); and satisfaction, usability, perceived quality, acceptability, and time and cost investments of PrEP care.

Furthermore, to investigate how suitable the offered PrEP care modalities are for participants, we will study the associations of the sociodemographic, behavioral, and psychosocial predictors of PrEP adherence with all outcomes mentioned above, and assess whether these associations vary between study arms. More specifically, we will study the associations of unprotected acts with problematic alcohol and drug use, risk perception of acquiring STIs and HIV infection, and sexual compulsivity. Adequate PrEP adherence is key to its effectiveness in preventing HIV, and regular PrEP adherence counseling is therefore recommended. While ample evidence exists on the facilitators and barriers of medication adherence across different populations and settings [43] (eg, medication for the prevention and treatment of cardiovascular diseases [44], chronic disease management [45], and HIV treatment [46]), this remains an understudied topic for MSM who use PrEP. We are interested in how PrEP intake is integrated in daily life, what circumstances may lead to inadequate PrEP adherence, and whether these factors are associated with fewer contact moments between health care providers and PrEP users. We will therefore study the relationship between habit formation and PrEP intake. We will also analyze mental well-being (eg, positive and negative emotions, depression, stress, and anxiety) in relation to adherence and unprotected acts, and explore the mediating effects of personality-related variables (eg, emotion regulation, coping skills, conscientiousness, and resilience). Finally, we will investigate to what extent having a social environment, which is measured by social norms, loneliness, and support systems, is related to PrEP adherence and retention in PrEP care.

### Study Setting

The EZI-PrEP study will be embedded in the government-funded National PrEP Pilot Program (NPP) that plans to run from August 2019 until July 2024 [47]. This program provides PrEP care to persons at high risk for HIV infection, notably MSM and TGDP, and is implemented by the sexual health centers (SHCs) of the public health service (PHS) regions throughout the Netherlands. The PrEP care offered consists of in-clinic monitoring visits every 3 months that are free of charge. PrEP tablets are provided at a reduced price of €7.50 (US $7.88) per 30 tablets (paid out-of-pocket by the PrEP user) and are provided only to those completing a monitoring visit. Following the Dutch PrEP guidelines applicable at the time when the study started (ie, April 15, 2019 [10]), each PrEP monitoring visit includes screening for HIV infection, bacterial STIs, and HCV infection, and, every 6 months, screening for kidney function (Table 1). It also includes counseling based on motivational interviewing techniques to stimulate informed and conscious decision-making by PrEP users in relation to their own sexual health, PrEP intake, and usage of other HIV-prevention strategies. PrEP naïve people are allowed to commence PrEP immediately after screening and counseling, and have to return to the SHC after the first month of PrEP use for repeat HIV testing. Three months after commencing PrEP, routine PrEP care begins with the first in-clinic monitoring visit (performed every 3 months). Individuals are allowed to test for HIV, STIs, and HCV free of charge in between regular PrEP monitoring visits at their own initiative. Individuals can freely choose and switch between daily or event-driven PrEP regimens. These guidelines have since been updated in 2022, with the most relevant modification being that the monitoring frequency may be adapted under certain individual cases [11].

**Table 1:** Overview of testing for HIV infection, sexually transmitted infections, hepatitis C virus infection, and kidney function as part of routine pre-exposure prophylaxis care for each EZI-PrEP study visit at sexual health centers in the Netherlands (based on Dutch national pre-exposure prophylaxis guidelines; April 19, 2019).

Laboratory measurement	Inclusion	Months								
		1	3	6	9	12	15	18	21	24
HIV	✓	✓^a^	✓	✓	✓	✓	✓	✓	✓	✓
Syphilis	✓		✓	✓	✓	✓	✓	✓	✓	✓
*Chlamydia trachomatis*	✓		✓	✓	✓	✓	✓	✓	✓	✓
*Neisseria gonorrhoeae*	✓		✓	✓	✓	✓	✓	✓	✓	✓
HCV^b^	✓		✓	✓	✓	✓	✓	✓	✓	✓
Creatinine	✓			✓		✓		✓		✓
Proteinuria	✓			✓		✓		✓		✓

^a^Only pre-exposure prophylaxis starters and restarters have a pre-exposure prophylaxis monitoring visit at month 1.

^b^HCV: hepatitis C virus.

In the Netherlands, PrEP can also be obtained through a general practitioner, although, in this instance, neither PrEP tablets nor laboratory testing are fully reimbursed by the Dutch health insurance. The Dutch insurance system has an obligatory deductible excess, meaning that the first €385 (US $404) of health care costs, including laboratory testing ordered by primary care providers, are paid for out-of-pocket. Moreover, knowledge and willingness to prescribe PrEP varies across general practitioners. Most people who are using or want to use PrEP in the Netherlands therefore procure PrEP through the NPP. This program has been capped at 8500 PrEP users, which has resulted in individuals being placed on waiting lists to obtain PrEP from the NPP [24].

### Study Design

**Figure 1:**
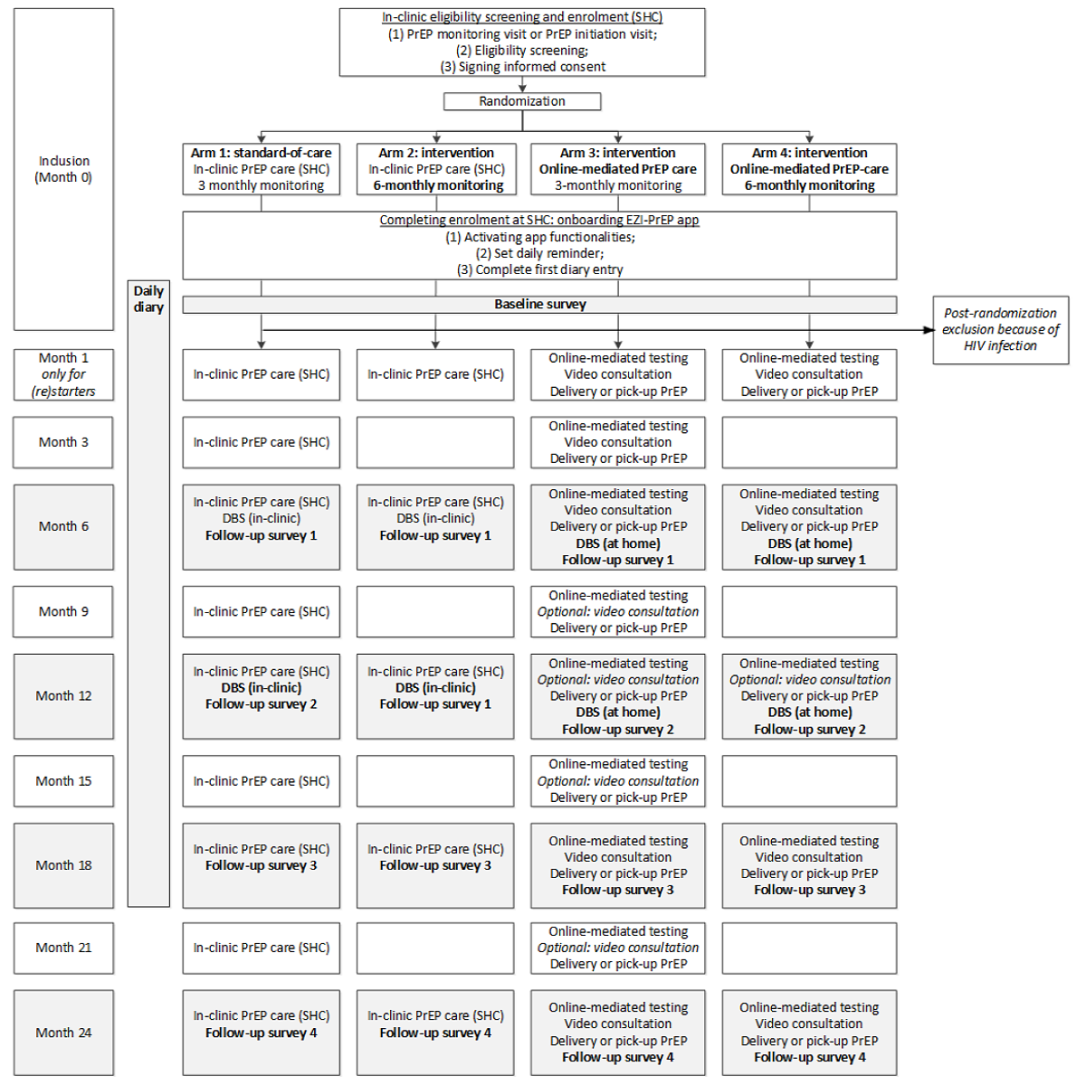
EZI-PrEP study flowchart. DBS: dried blood spot; EZI-PrEP: E-Health for Zero Infections - facilitating access to and use of Pre-Exposure Prophylaxis in the Netherlands; PrEP: pre-exposure prophylaxis; SHC: sexual health center.

**Figure 2:**
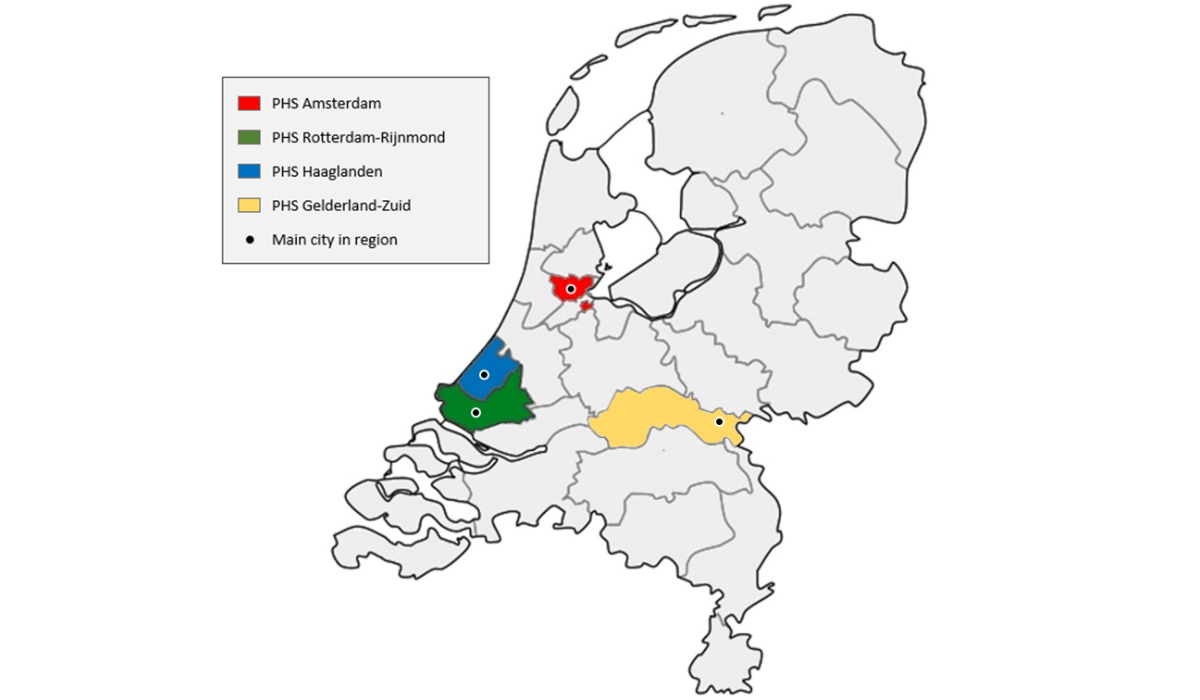
Map of the Netherlands with EZI-PrEP study sites. EZI-PrEP: E-Health for Zero Infections - facilitating access to and use of Pre-Exposure Prophylaxis in the Netherlands; PHS: public health service.

The EZI-PrEP study will be a 2×2 factorial, 4-arm, open-label, multi-center, randomized, controlled, noninferiority trial. Study participants will be randomized to 1 of 4 arms: (1) in-clinic monitoring every 3 months (standard of care), (2) in-clinic monitoring every 6 months, (3) online monitoring every 3 months, or (4) online monitoring every 6 months (Figure 1). The study will be implemented at SHCs in Amsterdam, Rotterdam-Rijnmond, Haaglanden, and Gelderland-Zuid (Figure 2). For each individual participant, the planned observation time is 24 months, starting after eligibility screening and enrollment.

All study participants will receive access to a smartphone app (the “EZI-PrEP app”), which contains a diary. Participants will be required to complete this diary daily during the first 18 months of the study period. This information will be used to measure the primary outcome (ie, unprotected acts). DBSs will be collected at months 6 and 12. Routine clinical data registered during PrEP monitoring visits and any additional STI testing or treatment visits from inclusion until the final monitoring visit at month 24 will be retrieved from the national STI surveillance system. Participants will be requested to complete 5 questionnaires (at baseline and at 6, 12, 18, and 24 months) to collect data on PrEP intake; sexual behavior; psychosocial characteristics; and the experiences, acceptability, and usability of the interventions. We will extract automatically registered data on app usage from the app.

### Study Population

We aim to include 442 individuals who are already using PrEP through the Dutch NPP or will be starting or restarting PrEP. Individuals starting PrEP are PrEP naïve (herein referred to as “PrEP starters”), whereas individuals restarting PrEP are persons who have used PrEP more than 6 months prior to inclusion and will recommence PrEP at inclusion (herein referred to as “PrEP restarters”). Both groups of individuals need to have a monitoring consultation after 1 month of starting or restarting PrEP as part of standard-of-care procedures and are therefore grouped in the protocol as “PrEP starters or restarters.” Our intention is that at least 25% of study participants will be PrEP starters or restarters.

The eligibility criteria will be as follows: (1) being eligible for the Dutch NPP (Textbox 1) [45]; (2) being 18 years or older; (3) living in the catchment area of a participating PHS; (4) being able to complete informed consent, medical history, the daily diary, and questionnaires in English or Dutch; (5) having an email address; (6) owning an iOS or Android mobile phone capable of running the study app; (7) having daily access to an internet connection; (8) being able to make online payments; and (9) providing informed consent. The exclusion criteria will be as follows: (1) having an HIV infection; (2) being advised by a physician to wait with starting or restarting PrEP (PrEP starters or restarters) or being advised to stop with PrEP (current PrEP users) for more than 1 month, such as in the case of decreased kidney function with an estimated glomerular filtration rate (eGFR) of less than 60 mL/min/1.73m^2^ or other kidney problems, or chronic use of medication that interacts with TFV/FTC; (3) being advised to have in-clinic monitoring every 3 months, such as in the case of adverse mental health conditions or life circumstances, chronic or acute hepatitis B virus infection, or other medical conditions that require special attention when using PrEP or may need a referral, such as osteoporosis or other bone diseases; and (4) participating in another clinical trial or research project related to HIV prevention.

**Eligibility criteria of the Dutch national pre-exposure prophylaxis pilot program (Dutch national pre-exposure prophylaxis guidelines dated April 15, 2019).**

Men who have sex with men or transgender and gender diverse people who in the 6 months prior to the pre-exposure prophylaxis (PrEP) request or PrEP consultation:

- Had anal sexual intercourse without a condom with a male partner having an unknown HIV status
- Had anal sexual intercourse without a condom with a male partner having a known HIV-positive status and a detectable viral load
- Was diagnosed with a rectal sexually transmitted infection or syphilis
- Received a prescription for HIV postexposure prophylaxis

### Recruitment

We will recruit participants mainly at the SHCs of the participating PHSs. Current PrEP users will be recruited during regular PrEP monitoring visits by a health professional (usually a nurse or a physician). Individuals who are starting or restarting PrEP will be recruited from the waiting lists for PrEP care via email, SMS text messaging, or a phone call and routine communication channels of the respective SHC. The enrollment visit will be scheduled together with the first PrEP consultation. To support the recruitment efforts of the SHCs, we developed a website [48] and promotional materials for distribution to SHC clients. In addition, depending on the recruitment rate in the participating SHCs, we plan to conduct 3 advertising rounds on the MSM dating platform Grindr, targeting potential participants living in the catchment areas of the 4 participating PHSs. All promotional materials and advertisements will link to the EZI-PrEP website page with a short application form.

### Enrollment Procedures

In preparation of the enrollment visit, potential participants will receive an email with the patient information folder and will be asked to download the EZI-PrEP study app. The enrollment interview will begin with a regular PrEP consultation at the SHCs, which will be combined with eligibility screening (Figure 1). Noneligible individuals will continue or commence routine PrEP care. Eligible individuals will be guided through study procedures and asked to sign an informed consent form, and will be subsequently randomized to 1 of the study arms. Each participant will then onboard the EZI-PrEP app and complete the diary for that day to ensure understanding of the questions asked in the diary. This first diary entry will not be used for analysis because it is completed in the presence of a health care worker. The enrollment interview will be finalized with scheduling of the next monitoring appointment and PrEP provision.

The Dutch PrEP guidelines allow for combining STI and HIV test specimen collection with PrEP dispensing, as long as a client can be easily reached to communicate HIV and STI test results [10,11]. In case of a positive HIV test result, the PrEP user will be notified immediately to discontinue their PrEP and will be referred to HIV care. This strategy may lead to postrandomization exclusion from the EZI-PrEP study within 1 week after randomization.

### Randomization

Participants will be randomized (stratified by PHS and by PrEP experience, that is, those starting or restarting PrEP, and current PrEP users) to 1 of 4 study arms in permuted blocks of 4. Randomization will be performed by a statistician of PHS Amsterdam who is not part of the EZI-PrEP study team at initiation of the study, using Stata Intercooled 15.1 (StataCorp). Sequentially numbered and colored nontransparent envelopes will be made, containing a code for the study arm. Each PHS will receive 2 sets of envelopes, red ones for those starting or restarting PrEP and blue ones for current PrEP users. During the enrollment interview, in the presence of the participant and dependent on their PrEP experience, the health care provider will open the next envelope to reveal the study arm and study number.

### Interventions

#### PrEP Care Every 6 Months

Participants in arms 2 and 4 (Figure 1) will receive PrEP care every 6 months. All study participants, irrespective of study arm, will be allowed to get screened free of charge for HIV and STIs in between scheduled PrEP monitoring visits (eg, after a partner notification for an STI or in case of STI-related symptoms). To allow for monitoring in between testing, we will ask participants to visit the SHC where they receive PrEP care.

#### Online PrEP Care

**Figure 3:**
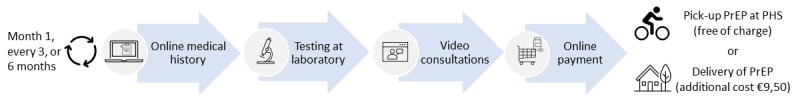
Process of online-mediated EZI-PrEP care. It should be noted that 6 months after inclusion, the video consultations will become optional, and PrEP provision will then be based on online (self-reported) medical history and test results. A currency exchange rate of €1=US $1.05 is applicable. EZI-PrEP: E-Health for Zero Infections - facilitating access to and use of Pre-Exposure Prophylaxis in the Netherlands; PHS: public health service; PrEP: pre-exposure prophylaxis.

Participants in arms 3 and 4 (Figure 1) will receive online-mediated PrEP care that mimics in-clinic PrEP care. Figure 3 depicts the process of online-mediated PrEP care. First, participants will access an established online HIV and STI test request platform for MSM called Testlab [49]. On this platform, participants will complete standard intake questions for a PrEP consultation. The number of PrEP tablets needed until the next monitoring date will also be reported. All medical information will be stored in the electronic patient files. Participants reporting STI symptoms at the scheduled online monitoring visit will be asked to visit the clinic for physical examination and treatment.

The participant will then receive a lab order from the Testlab platform to visit one of the connected laboratories to provide samples for HIV infection, STI, HCV infection, and kidney function testing (Table 1). Negative test results for HIV infection, STI, and HCV infection; normal kidney function test results; and positive test results for bacterial STIs will be communicated via the Testlab platform. Treatment of bacterial STIs will occur in the clinic at SHCs. In case of a positive test result for HIV or HCV, or a decreased kidney function, participants will receive a phone call to make an appointment on the same day.

To provide counseling on sexual well-being, PrEP intake, and other HIV prevention strategies, a video consultation will take place. These video consultations will become optional after 6 months. The continuation of video consultation will be based on shared decision-making between the participant and the SHC health care provider. Self-reported medical history and testing for HIV infection, STIs, HCV infection, and kidney function will remain mandatory for PrEP provision. For the purpose of the study, a mandatory video consultation will take place at 18 and 24 months to inform participants about study procedures that change at those time points (Figure 1).

Participants in the online arms may opt for home delivery of PrEP tablets, for which a service fee of €9.50 ($9.98) will be requested and will require an online payment. Pick up of PrEP tablets from the SHC will also be possible without any additional cost. The SHC physician will determine the number of tablets that a participant is able to obtain based on the intended PrEP regimen and the number of tablets requested by the participant in the self-reported medical history.

### EZI-PrEP Applications

#### EZI-PrEP App

**Figure 4:**
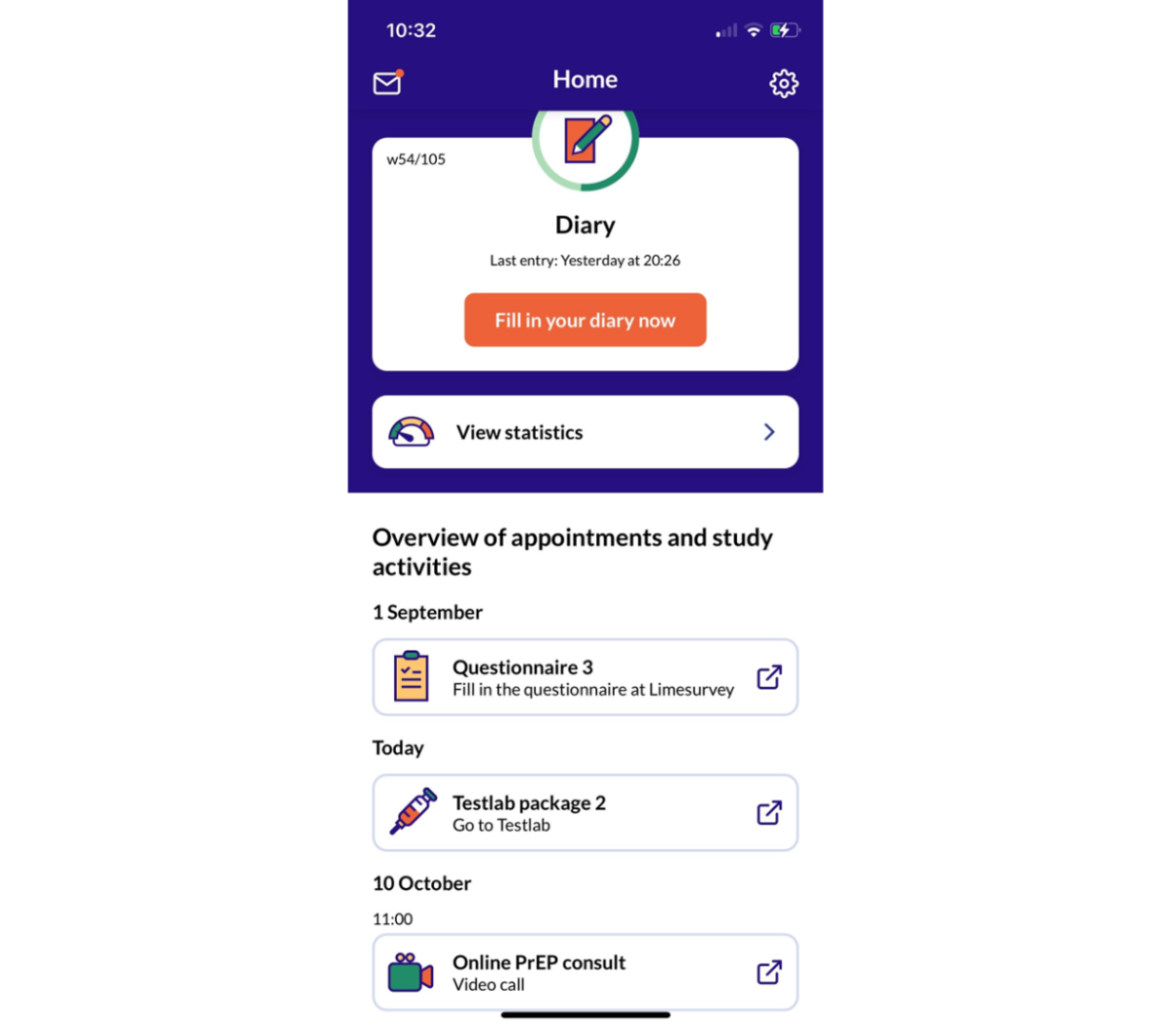
Homepage of the EZI-PrEP app. It should be noted that Testlab package 2 and online PrEP consultation are only visible to EZI-PrEP participants in arms 3 and 4. Arm 3: online monitoring every 3 months; Arm 4: online monitoring every 6 months; EZI-PrEP: E-Health for Zero Infections - facilitating access to and use of Pre-Exposure Prophylaxis in the Netherlands; PrEP: pre-exposure prophylaxis.

All participants will get access to the free-of-charge, password-protected EZI-PrEP app through the Apple and Google Play stores. App functionalities will depend on their allocated mode of PrEP care provision. All participants will have access to the daily diary, the summary of data entered in the diary, and a timeline with an overview of upcoming PrEP monitoring visits and study questionnaires (Figure 4). Participants receiving online PrEP care will also receive access to the online-mediated test request platform, video consultation software, and PrEP payment module. All functionalities will be immediately accessible by clicking on the activity in the timeline of the app via a single sign on. All participants will be able to receive messages in the app from SHC health care workers and study staff, but will not be able to send messages via the app (Figure 4).

#### EZI-PrEP Administration Portal

The EZI-PrEP app and all functionalities, except for the diary, will be controlled by SHC health care workers and EZI-PrEP study staff via a web portal that uses a cloud platform to exchange data with the EZI-PrEP app. The administration portal will be used to activate participant accounts, with functionalities enabled according to the study arm allocation. The portal will include an event planner to register the date and time of in-clinic visits, online-mediated testing, and video consultations, and activate the PrEP payment module upon entering the number of tablets that can be obtained by the online participants. The portal will also be used for scheduling questionnaires and, for online participants only, to activate home delivery of a DBS home sampling kit. In-app messages will be sent via the administration portal as well. Health care workers will be able to only access the accounts of participants receiving PrEP care at their SHCs.

### Laboratory Procedures

Laboratory procedures will be performed according to routine STI and PrEP care clinical protocols, which may slightly differ across study sites. A detailed description of the laboratory procedures at each study site can be found in Multimedia Appendix 1.

### Data Collection Methods

#### EZI-PrEP Diary

To minimize recall bias and increase the accuracy of self-reported PrEP intake, casual anal sex, and condom use, participants will be requested to complete a daily diary for primary outcome measurement [50]. Completing the diary may be burdensome for certain participants and could result in less motivation to remain in the study. Minimizing the length of the diary collection period could help maintain sufficient motivation to complete the diary and continue study participation. During the first 18 months of the study, each participant will be completing 4 to 6 daily questions. These diary questions ask about PrEP use, sexual behavior of the previous day, and current mood. The first question (“Do you use daily or event-driven PrEP?”) will be asked once and set as default (ie, prefilled) until the participant changes to a different regimen, which can be selected in a new diary on the following day. The second question (“How many PrEP pills did you take?”) will need to be answered with 0, 1, or 2. In case of 1 or 2 tablets, the third question will be prompted (“At what time did you take PrEP?”), which will need to be answered with a specific time. The fourth question (“Did you have anal sex with a casual partner?”) will need to be answered with yes or no. If yes is answered, the fifth question (“Did you use a condom every time?”) will be prompted, which will need to be answered with yes or no. The sixth question (“How do you feel today?”) will need to be answered on a discrete scale with 5 options, ranging from 1 (depicted by a red sad face) to 5 (a green happy face). Participants will be allowed to change their answers until automatic submission of the diary when the final question is answered. The average expected duration of completing the daily diary is 90 seconds.

The diary that is completed on a given day will ask about PrEP intake and sexual behavior of the day before, which is indicated on the top of the screen. If a participant forgets to enter their information in the daily diary, they will be allowed to retrospectively fill in information, but only for the past 2 days.

To increase diary completion adherence, we will send automated daily notifications at 10 AM Central European Time (CET); however, it will be possible to deactivate this reminder in the settings of the app. If a participant has not completed their diary for 7 days, an email with a reminder and explanation of the importance of the diary for the study will be sent. After 14 days, another reminder that the diary is crucial for the study will be sent to the participant and to the PHS where they are under care, so that the importance of diary completion can be discussed in the upcoming monitoring visit. Being unable to complete the diary will not be considered as a reason to end participation in the study.

#### DBS Collection

We will collect DBSs to measure intracellular concentrations of TFV-DP and emtricitabine-triphosphate (FTC-TP). Blood will be spotted on Whatman 903TM Protein Saver Cards (GE Healthcare Bio-Sciences Corp). For participants receiving in-clinic PrEP care, study nurses will collect whole blood via phlebotomy and spot this onto the DBS card during the 6- and 12-month PrEP monitoring visits. Participants receiving online-mediated PrEP care will receive a DBS home sampling kit after completing their PrEP monitoring visit at months 6 and 12. This kit contains the DBS card with 5 spots, 2 contact-activated lancets (2.0-mm BD Microtainer), an alcohol wipe, a gauze wipe, 2 band aids, a grip seal bag, a desiccant sachet with a humidity indicator (MiniPax, Multisorp Technologies), a preposted envelope, and paper instructions with a QR code to an instruction video. Participants will be requested to send the completed DBSs in the preposted envelope to the Amsterdam regional microbiology laboratory. All DBS cards will be stored at −20°C at the Amsterdam regional microbiology laboratory and shipped in batches for analysis to Skaggs School of Pharmacy and Pharmaceutical Sciences (University of Colorado Anschutz Medical Campus). Owing to financial constraints, only DBS cards from daily PrEP users will be analyzed. The procedure to measure TFV-DP and FTC-TP in DBSs has been described previously [51]. The results will not be reported back to the participants. For participants with incident HIV infection, the most recent DBS prior to infection will be analyzed for TFV-DP, FTC-TP, and HIV RNA

#### Clinical Data

We will obtain the clinical data of all PrEP and STI consultations of study participants between enrollment and study completion from the Dutch national SHC surveillance database (SOAP). During every consultation at the SHC, data are collected on the following: (1) sociodemographic characteristics (age, gender, highest educational level, country of birth, and country of birth of parents); (2) sexual behavior and STI/HIV risk factors (number of sex partners, condom use, group sex, use of alcohol and drugs during sex, sex work, having been a victim of sexual violence, and sex partners from STI or HIV endemic countries); (3) reason for consultation (partner notification, STI-related symptoms, PrEP, and STI testing); (4) PrEP use (history of PrEP use, reasons for stopping PrEP, and number of PrEP tablets provided); and (5) HIV infection, STI, and HCV infection testing and diagnosis. Data on kidney function and proteinuria will be retrieved from the electronic patient files.

#### Study Questionnaires

The first 4 questionnaires (ie, at baseline, and 6, 12, and 18 months) will ask about PrEP intake, sexual behavior, risk perception related to STIs and HIV infection, and STI testing at facilities other than SHCs. We will include several psychosocial measurements in the questionnaires (summarized in Table 2). The questionnaire at month 12 will include questions on social norms with respect to PrEP use, loneliness, and the presence of social support systems. To investigate the usability and acceptability of the PrEP care modality, we will include questions based on Davis’ model of technology acceptance [52] at months 6, 12, and 18. These questionnaires will also include questions regarding experiences with PrEP care and to what extent participants felt supported in their PrEP use. To assess the usability and acceptability of online-mediated PrEP care specifically, we will include the system usability scale [53] at month 18 for online study participants. We will also ask them to rate the different steps during their online PrEP care. The fifth (and last) questionnaire, at month 24, will focus on the quality of PrEP care within the EZI-PrEP study and in the Netherlands in general, as well as the time and cost investments of study participants in receiving PrEP care.

**Table 2:** Overview of psychosocial measurements within the EZI-PrEP study in each survey round.

Scale	Abbreviation	Aspect measured	Baseline survey (month 0)	Follow-up survey			
				Month 6	Month 12	Month 18	Month 24
Self-reported habit index	SHI	Habit formation	✓	✓	✓	✓	
Patient Health Questionnaire-9	PHQ-9	Severity of depressive symptoms	✓	✓	✓	✓	
Generalized Anxiety Disorder Assessment	GAD-7	Generalized anxiety disorder	✓	✓	✓	✓	
Sexual compulsivity scale	SCS	Sexual compulsivity	✓		✓		
Alcohol Use Disorders Identification Test	AUDIT	Problematic alcohol use	✓			✓	
Drug Use Disorders Identification Test	DUDIT	Problematic drug use	✓			✓	
Perceived stress questionnaire	PSQ20	Perceived stress		✓	✓	✓	
Brief Resilience Scale	BRS	Resilience		✓			
Cognitive Emotion Regulation Questionnaire	CERQ-short	Coping skills			✓		
HEXACO^a^ Personality Inventory-Revised	HEXACO-PI-R	Conscientiousness			✓		

^a^HEXACO is an acronym for “Honesty-humility,” “Emotionality,” “Extraversion,” “Agreeableness,” “Conscientiousness,” and “Openness to experience.”

### Data Analysis

#### Primary Outcome

The primary outcome (“unprotected act”) will be assessed using daily self-reported data on sexual behavior, pill intake, and condom use. We will use all available data, meaning that observation time and all data of those who are lost to follow-up or have dropped out will be included until their last day in the study. We will determine, for each day of follow-up, whether a condomless anal sex act with a casual partner took place. We will also determine whether sufficient PrEP pills were taken around the time of this condomless anal sex act. Using this information, the condomless anal sex act will be considered as either unprotected or not. We will then calculate the total number of unprotected acts (n) and the total person-years of observation. Incidence rates per 100 person years will be calculated by dividing the sum of all unprotected acts by the sum of person-years of observation and multiplying the result by 100. We will calculate the incidence rates among participants of online PrEP care (arms 3 and 4), in-clinic PrEP care (arms 1 and 2), monitoring every 6 months (arms 2 and 4), and monitoring every 3 months (arms 1 and 3). Two main analyses will be conducted in which we will test the ratio of the incidence rates of unprotected acts for (1) online PrEP care versus in-clinic PrEP care and (2) monitoring every 6 months versus monitoring every 3 months. The analyses will be based on crude unadjusted incidence rate ratios.

A noninferiority trial intends to demonstrate that one intervention is not unacceptably worse than a comparison intervention [54]. To do so, the lower limit of the 95% CI (or 2-sided 97.5% CI) should not extend beyond a predefined margin, which is referred to as the noninferiority margin. In this study, noninferiority will be tested using a 1-sided test based on maximum likelihood estimators with a type 1 error at 0.0125 (ie, with 2 comparisons using Bonferroni corrections=0.025/2) [40] and a noninferiority ratio of 1.8. If the upper 2-sided 97.5% CI of the incidence rate ratio is below 1.8, we will conclude that the intervention arms are noninferior to the comparator arms.

To determine whether the incidence rates change over time, we will model the number of unprotected acts as a function of 1-month intervals using Poisson regression (or negative-binomial regression, if the standard deviation is far greater than the mean of the outcome, ie, overdispersion) and test for changes in time using a Wald χ^2^ test. We will test for the interaction between time and intervention arm.

#### Secondary Outcomes

We will assess intracellular concentrations of TFV-DP and FTC-TP using DBSs collected at month 12 in all participants using daily PrEP. The lower limit of detection is determined as 25 fmol/punch and 50 fmol/punch for TFV-DP and FTC-TP, respectively. The distribution of the intracellular concentrations of TFV-DP and FTC-TP will be compared between daily PrEP users in (1) online PrEP care versus in-clinic PrEP care and (2) monitoring every 6 months versus monitoring every 3 months, using a rank-sum test. In a secondary analysis, the log-transformed values will be compared using a linear regression.

The incidence rates of HIV infection and TFV/FTC-resistant HIV infection, and the incidence rates of bacterial STIs (ie, chlamydia, gonorrhea, and syphilis), HCV infection, and eGFR of less than 60 mL/min/1.73m^2^ will be assessed. The incidence rates are calculated by dividing the number of diagnoses by the person-years of observation. The incidence rates will be compared for (1) online PrEP care versus in-clinic PrEP care and (2) monitoring every 6 months versus monitoring every 3 months.

Retention in PrEP care is defined as having had a PrEP prescription no longer than 8 months ago. Participants will be considered lost to follow-up when they do not complete a PrEP monitoring visit at month 18 within 2 months of the planned date. They will be regarded as lost to follow-up from the date of the last contact with the study (ie, completed a diary, questionnaire, or consultation). We will compare proportions of participants who are retained in PrEP care or are lost to follow-up for (1) online PrEP care versus in-clinic PrEP care and (2) monitoring every 6 months versus monitoring every 3 months, using the Pearson χ^2^ test.

Satisfaction with PrEP care will be compared between study arms. Furthermore, we will analyze how usability of the online PrEP service differs between online-mediated PrEP care every 3 months and every 6 months. For these analyses, we will use the Wilcoxon signed-rank test of the Likert score outcomes.

We will investigate the associations between sociodemographic and psychosocial determinants and the incidence rates of unprotected acts; HIV infection, STIs, and HCV infection; retention in PrEP care; and loss to follow-up. A wide range of parameters, mentioned in the Data Collection Methods section, will be included in our analyses. The analyses will involve logistic models, Cox proportional hazards models, and Poisson regression models.

### Power and Sample Size Calculation

We assume that the primary outcome measure of unprotected acts will be Poisson distributed with the total number per 100 person-years given as an incident rate (λ) and the total time during monitoring (*t*). We define the incidence rate in the experimental arm as λ_e_ and the incidence rate in the control arm as λ_c_. We aim to reject the null hypothesis that the ratio λ_e_/λ_c_ is greater than or equal to a predetermined noninferiority ratio (r). We consider that a noninferiority ratio of 1.8 is able to preserve clinically acceptable noninferiority bounds with increasing λ.

In a previous PrEP study in the Netherlands (Amsterdam PrEP demonstration project [“AMPrEP study”]), the overall incidence rate of unprotected acts was 78/100 person-years [50]. The AMPrEP study included participants with a high number of sex partners and very high incidence of bacterial STIs. Participants of the NPP have reported fewer casual sex partners and have a lower incidence of bacterial STIs [55]. The EZI-PrEP participants are therefore expected to have fewer unprotected sex acts than the AMPrEP participants. We expect the incidence rate of unprotected acts in the experimental arm (λ_e_) and control arm (λ_c_) to be at least 36/100 person-years. We also assume a median monitoring time of 18 months. Using a Bonferroni-corrected 1-sided type 1 error of 0.0125 (=0.025/2 for a total of 2 comparisons), we will need 368 participants to have 90% power to demonstrate noninferiority using the test statistic based on maximum likelihood estimators from Stucke and Kieser [56]. If λ is higher than 36/100 person-years, we will have sufficient power to demonstrate noninferiority. Considering expected loss to follow-up, we have increased the target sample size by 20%, and thus, the total required sample size is 442.

### Ethical Considerations

This study has been approved by the medical ethics committee of the Amsterdam University Medical Centers (file number 2020_154 - NL74494.018.20). The trial has been registered at ClinicalTrials.gov (number NCT05093036).

## Results

The results of the primary outcome assessment are expected by Fall 2024. Completion of the 24-month questionnaire and retrieval of STI data are expected by Fall 2024. Analysis of secondary outcomes will be conducted in 2024 and 2025.

## Discussion

We have designed the EZI-PrEP study in order to assess whether online-mediated PrEP care and a reduced frequency of monitoring are noninferior compared to standard practice. In this study, we will develop, implement, and rigorously evaluate an online-mediated PrEP care service and a less frequent monitoring schedule (every 6 months) among MSM and TGDP in 4 regions in the Netherlands. Studies in countries with concentrated HIV epidemics and PrEP programs for MSM show the potential of PrEP programs to reduce population-level HIV incidence [57,58]. Reducing the number of in-clinic visits may reduce the burden of PrEP care and might help increase uptake and improve retention. Additionally, it may improve access to PrEP as it may make more resources available and enlarge capacity at facilities providing PrEP. Online-mediated PrEP care and monitoring every 6 months are promising options to reduce in-clinic PrEP visits, but it is unclear how this affects PrEP adherence. Since both modalities involve less intense contact between PrEP users and health care providers, and thus less guidance in PrEP use, it could theoretically have an adverse effect on PrEP adherence. The EZI-PrEP study will be able to provide evidence on the efficacy and safety of online-mediated PrEP care and PrEP care every 6 months compared to standard in-clinic PrEP care every 3 months in terms of PrEP adherence as a proxy for HIV risk. If these services are found to be noninferior, both online access to PrEP and reduced monitoring frequency could become viable evidence-based PrEP care options.

The EZI-PrEP study will contribute crucial data to the ongoing debate on optimal HIV and STI screening frequency for MSM who use PrEP. By comparing incidence rates of HIV infection, STIs, and HCV infection between the 3-month and 6-month monitoring study arms, we will provide evidence on the impact of testing every 6 months on STI diagnosis and treatment. If noninferiority in terms of PrEP adherence is concluded and STI incidence rates are similar between the 6-month and 3-month monitoring arms, a stronger case can be made to offer PrEP monitoring every 6 months as the standard of care. If STI incidence rates are not similar between the study arms, we will be able to examine to what extent this is related to particular sociodemographic, sexual behavior, or psychosocial factors and advise on implementation accordingly. EZI-PrEP study participants will be allowed to test for HIV, STIs, and HCV in between regular PrEP monitoring visits at their own initiative. The EZI-PrEP study will also be able to quantify the extent to which in-between visits occur among PrEP users receiving monitoring every 6 months.

Since adherence to the PrEP intake regimen is crucial for its effectiveness [3,5-7], having a thorough understanding of enabling and disenabling factors of PrEP adherence is important. The EZI-PrEP study will provide new insights on the associations between sociodemographic and psychosocial variables on the one hand and PrEP adherence on the other hand. Insights into the role of such sociodemographic and psychological factors in the context of adherence may be used to further improve the content or conduct of PrEP consultations and inform interventions that support PrEP adherence. Furthermore, we will investigate the relationship of these factors with PrEP adherence, STIs, retention, and satisfaction with PrEP care, according to the study arm. The results of these analyses could be used to determine if these new PrEP care modalities should be implemented widely or should be offered to specific subpopulations.

The main methodological challenge of this study is the measurement of the primary outcome measure, which is based on self-reported PrEP intake and sexual behavior. In the AMPrEP study, it was found that reporting of daily data decreases over time, which may confront us with a substantial amount of missing data. We will therefore implement an intensive scheme of reminders. Additionally, the diary in itself may cause behavioral changes (ie, Hawthorne effect). For instance, PrEP intake may be affected due to the daily reminder to complete the diary. Completing the diary on a daily basis might also be too burdensome for some participants, and as a result, their motivation to complete the study could be low. However, because of randomization, any effect of daily diary entry on behavior or participation is not expected to differ across the 4 arms. A major strength of our study is that it is embedded in routine PrEP care, which increases the generalizability of study outcomes and will provide a realistic perspective on the implementation of 2 new PrEP care modalities. If these modalities are found to be noninferior, they can be easily implemented in routine care using the workflows developed in this study and applied to the 4 participating PHSs. Furthermore, combining monitoring every 6 months and online-mediated PrEP care in this trial offers a unique opportunity to study real-life implementation of these PrEP modalities.

In conclusion, the results of the EZI-PrEP trial will provide evidence on the noninferiority of online-mediated PrEP care compared to in-clinic care and the noninferiority of monitoring every 6 months compared to monitoring every 3 months in terms of PrEP adherence. It will also provide insights into screening every 6 months for HIV infection, STIs, and HCV infection among MSM who use PrEP, and thereby will contribute to the literature on the optimal STI screening frequency among PrEP users. Moreover, the study will investigate to what extent these PrEP care modalities are appealing to PrEP users in general and if particular PrEP users would benefit more from these modalities than others. Finally, the study will provide insights into the sociodemographic and psychosocial factors that influence PrEP adherence. The EZI-PrEP study aims to inform policymakers and sexual health centers in deciding which strategies would be appropriate for PrEP care. The findings will contribute to accessible, low-cost, scalable, and sustainable PrEP care.

## Appendix

Multimedia Appendix 1 Laboratory procedures at each study site.

## Abbreviations

AMPrEP: Amsterdam PrEP demonstration project
DBS: dried blood spot
eGFR: estimated glomerular filtration rate
EZI-PrEP: E-Health for Zero Infections - facilitating access to and use of Pre-Exposure Prophylaxis in the Netherlands
FTC: emtricitabine
FTC-TP: emtricitabine-triphosphate
HCV: hepatitis C virus
MSM: men who have sex with men
NPP: National PrEP Pilot Program
PHS: public health service
PrEP: pre-exposure prophylaxis
SHC: sexual health center
STI: sexually transmitted infection
TFV: tenofovir
TFV-DP: tenofovir-diphosphate
TGDP: transgender and gender diverse people

## Data Availability

Data sharing is not applicable to this article as no data sets have been generated or analyzed.
