# Supplementary material for: Online-Mediated HIV Pre-exposure Prophylaxis Care and Reduced Monitoring Frequency for Men Who Have Sex With Men: Protocol for a Randomized Controlled Noninferiority Trial (EZI-PrEP Study)
Source: JMIR Res Protoc. 2023 Nov 8;12:e51023. doi: 10.2196/51023 (PMC10666015; doi:10.2196/51023)
Supplement: Multimedia Appendix 1 [file resprot_v12i1e51023_app1.docx]

**Multimedia Appendix 1.** Laboratory procedures at each study site.

*Public Health Service of Amsterdam (Amsterdam regional microbiology laboratory, Amsterdam)*

HIV antigens and antibodies will be tested using the LIAISON XL Murex HIV Ag/Ab assay (Diasorin, Saluggia, Italy) with immunoblot confirmation (INNO_LIA HIV I/II Score; Fujirebio, Ghent, Belgium). Serology testing for syphilis will be done using the LIAISON Treponema Screen assay (DiaSorin, Saluggia, Italy), and VDRL (RPR-Nosticon II; bioMérieux, Marcy-l'Étoile, France). Urine, anal and pharyngeal swabs will be tested for *Chlamydia trachomatis* and *Neisseria gonorrhoeae* using nucleic acid amplification testing (Aptima Combo 2 assay; Hologic, San Diego, California, USA). HCV-naïve participants will be tested for HCV antibodies (Liaison XL HCV-Ab, DiaSorin, Saluggia, Italy) at inclusion and every 3 months during follow-up monitoring visits. HCV-naïve participants with newly detected HCV antibodies will be tested every 6 months during follow-up for HCV-RNA (Aptima HCV Quant Dx Assay, Hologic, San Diego, California, USA). Participants with a previous HCV antibody-positive test result will be tested for HCV-RNA (Aptima HCV Quant Dx Assay, Hologic, San Diego, California, USA) at inclusion and every 6 months during follow-up. Plasma creatinine results will be used to calculate the estimated Glomerular Filtration Rate (eGFR) using CKD-EPI Creatinine Equation (2021). Urine samples will be tested for proteinuria using a dipstick (Uricolor 10 teststrips for urine analyes proteine and glucose, Abbott, Chicago, Illinois, USA).

*Public Health Service of Rotterdam-Rijnmond (Erasmus MC laboratory, Rotterdam)*

HIV antigens and antibodies will be tested using the LIAISON XL Murex HIV Ag/Ab assay (Diasorin, Saluggia, Italy) with immunoblot confirmation (INNO_LIA HIV I/II Score; Fujirebio, Ghent, Belgium). Serology testing for syphilis will be done using the LIAISON Treponema Screen assay (DiaSorin, Saluggia, Italy), and VDRL (RPR-Gold Standard; Gold Standard Diagnostics, Davis , USA). Urine, anal and pharyngeal swabs will be tested for Chlamydia trachomatis and Neisseria gonorrhoeae using nucleic acid amplification testing (cobas® CT/NG, Roche Diagnostics, Basel, Switzerland). HCV-naïve participants will be tested for HCV antibodies (LIAISON® XL Murex HCV Ab, DiaSorin, Saluggia, Italy) at inclusion and every 3 months during follow-up monitoring visits. HCV-naïve participants with newly detected HCV antibodies will be tested every 6 months during follow-up for HCV-RNA (Aptima HCV Quant Dx Assay, Hologic, San Diego, California, USA). Participants with a previous HCV antibody-positive test result will be tested for HCV-RNA (Aptima HCV Quant Dx Assay, Hologic, San Diego, California, USA) at inclusion and every 6 months during follow-up. Plasma creatinine results will be used to calculate the estimated Glomerular Filtration Rate (eGFR) using CKD-EPI Creatinine Equation (2021). Urine samples will be tested for proteinuria using a dipstick (Siemens Multistix 5 (reagent strips for urine alysis).

*Public Health Service of Haaglanden* (Haaglanden Medical Centre, The Hague)

Serology tests will be altered during the study due to switch of apparatus. Until September 2022 HIV antigens and antibodies, syphilis screen and HCV antibodies will be tested using the Atellica system (Siemens, Erlangen, Germany) with HIV Ag/Ab, TP-screen and anti-HCV, respectively. After September 2022 screening for HIV, syphilis and hepatitis C was performed by Liaison XL (Diasorin, Italy) with murex HIV Ag/Ab assay, Treponema Screen assay and murex HCV-Ab Quant assay, respectively. Positive HIV screening was confirmed by immunoblot (INNO-LIA HIV I/II Score; Fujirebio, Ghent, Belgium) and/or single p24 antigen assay (VIDAS HIV P24 II; BioMerieux, Marcy-l’Étoile, France). Positive syphilis screen was followed by immunoblot confirmation (INNO-LIA Syphilis score; Fujirebio, Ghent, Belgium) and RPR testing (AIS RPR reditest; Arlington, Springville, UT, USA). Participants with an know positive status for both syphilis screen and immunoblot were subjected to RPR testing only. Positive HCV screening was confirmed by immunoblot (INNO-LIA® HCV Score; Fujirebio, Ghent, Belgium) as well as HCV-RNA detection (Untill May 2022 by Aptima HCV Quant Dx Assay, Hologic, San Diego, CA, USA; As of May 2022 by HCV VL; GeneXpert, Cepheid, Sunnyvale, CA, USA). Participants with a known positive HCV test-result, were tested for HCV-RNA only. Urine, anal and pharyngeal swabs will be tested for *Chlamydia trachomatis* and *Neisseria gonorrhoeae* using nucleic acid amplification testing (Aptima Combo 2 assay; Hologic, San Diego, California, USA). Indeterminate results and possible double infections were also tested by single target assays (Aptima Combo 2 assay; Hologic, San Diego, California, USA). Plasma creatinine results will be used to calculate the estimated Glomerular Filtration Rate (eGFR) using CKD-EPI Creatinine Equation (2021). Urine samples will be tested for proteinuria using a dipstick (Uricolor 10 teststrips for urine analyes proteine and glucose, Abbott, Chicago, Illinois, USA).

*Public Health Service of Gelderland – Zuid (Radboud UMC, Nijmegen)*

HIV screening will be performed using a ‘HIV-Combo’ chemiluminescence immunoassay (CLIA) (LIAISON XL Murex HIV Ag/Ab assay, Diasorin); positive samples undergo confirmation by immunoblot (Geenius™ HIV1/2; Bio-Rad). In case of remaining doubt, samples will be assessed by nucleic acid amplification testing (Xpert HIV-1 Viral load, Cepheid). Serological screening for syphilis will be performed using a Treponemal CLIA (the LIAISON Treponema Screen assay, DiaSorin), with confirmation by Treponema pallidum particle agglutination (SERODIA-TPPA, Fujirebio); disease activity will be assessed by rapid plasma reagin (Carbogen, Tulip Diagnostics). Urine, anal and pharyngeal swabs will be tested for Chlamydia trachomatis and Neisseria gonorrhoeae using nucleic acid amplification testing (Cobas CT/NG; Roche). HCV screening will be performed using a HCV CLIA (Liaison XL HCV-Ab, DiaSorin), with confirmation by immunoblot (Geenius™ HCV; Bio-Rad). Participants with a previous HCV antibody-positive test will be tested for re-infection by nucleic acid amplification testing (Xpert HCV Viral load, Cepheid). Plasma creatinine results will be used to calculate the estimated Glomerular Filtration Rate (eGFR) using CKD-EPI Creatinine Equation (2021). Urine samples will be tested for proteinuria using a dipstick (Combur3-Test for urine analyes proteine and glucose, Roche Diagnostics, Basel, Switzerland).
